# Vitexin suppresses autophagy to induce apoptosis in hepatocellular carcinoma via activation of the JNK signaling pathway

**DOI:** 10.18632/oncotarget.11731

**Published:** 2016-08-31

**Authors:** Jin-Dan He, Zhen Wang, Shi-Peng Li, Yan-Jie Xu, Yao Yu, Yi-Jie Ding, Wen-Li Yu, Rong-Xin Zhang, Hai-Ming Zhang, Hong-Yin Du

**Affiliations:** ^1^ First Central Clinical College, Tianjin Medical University, Tianjin 300192, P.R. China; ^2^ Department of Anesthesiology, Tianjin First Central Hospital, Tianjin 300192, P.R. China; ^3^ Department of Liver Transplantation, Oriental Organ Transplant Center of Tianjin First Central Hospital, Key Laboratory of Organ Transplantation of Tianjin, Tianjin 300192, P.R. China; ^4^ Department of General Surgery, The People's Hospital of Jiaozuo City, Jiaozuo 454002, P.R. China; ^5^ Laboratory of Immunology and Inflammation, Department of Immunology, Key Laboratory of Immune Microenvironment and Diseases of Educational Ministry of China, Basic Medical College, Tianjin Medical University, Tianjin 300070, P.R. China

**Keywords:** vitexin, hepatocellular carcinoma, autophagy, apoptosis, JNK signaling

## Abstract

Vitexin, a flavonoids compound, is known to exhibit broad anti-oxidative, anti-inflammatory, analgesic, and antitumor activity in many cancer xenograft models and cell lines. The purpose of this study was to investigate the antitumor effects and underlying mechanisms of vitexin on hepatocellular carcinoma. In this study, we found that vitexin suppressed the viability of HCC cell lines (SK-Hep1 and Hepa1-6 cells) significantly. Vitexin showed cytotoxic effects against HCC cell lines *in vitro* by inducing apoptosis and inhibiting autophagy. Vitexin induced apoptosis in a concentration-dependent manner, and caused up-regulations of Caspase-3, Cleave Caspase-3, and a down-regulation of Bcl-2. The expression of autophagy-related protein LC3 II was significantly decreased after vitexin treatment. Moreover, western blot analysis presented that vitexin markedly up-regulated the levels of p-JNK and down-regulated the levels of p-Erk1/2 in SK-Hep1 cells and Hepa1-6 cells. Cotreatment with JNK inhibitor SP600125, we demonstrated that apoptosis induced by vitexin was suppressed, while the inhibition of autophagy by vitexin was reversed. The results of colony formation assay and mouse model confirmed the growth inhibition role of vitexin on HCC *in vitro* and *in vivo*. In conclusion, vitexin inhibits HCC growth by way of apoptosis induction and autophagy suppression, both of which are through JNK MAPK pathway. Therefore, vitexin could be regarded as a potent therapeutic agent for the treatment of HCC.

## INTRODUCTION

Hepatocellular carcinoma (HCC), the fifth most common cancer worldwide, is characterized as having a poor prognosis and high mortality [1]. Liver transplantation (LT), hepatic resection, and early-stage radiofrequency ablation (RFA) are regarded as potentially curative therapies [2]. Palliative treatment includes transarterial chemoembolization (TACE), radiotherapy, systemic chemotherapy and targeted therapy with sorafenib [3]. However, the overall survival rate of patients with HCC remains low, even with these treatment. Recently, a number of natural extracts, containing taxol [4], vitexin [5] and evodiamine [6], have been evaluated for possible use in the treatment of cancer, thus providing new strategies for the research and development of antitumor agents.

Vitexin, (apigenin-8-C-D-glucopyranoside), a naturally-derived flavonoids compound, has been used as a traditional Chinese medicine for the treatment of a variety of diseases [7, 8]. The anti-oxidant and anti-inflammatory properties of vitexin have been shown to provide significant protective effects against myocardial ischemia reperfusion injury [9]. It has also been demonstrated that vitexin shows potential as an inhibitor of cancer cell growth within a variety of cancer cell lines such as breast, prostate, ovarian, and esophageal cancer as well as for choriocarcinoma, which appears to involve its capacity for apoptosis in cancer cells [10–12].

Apoptosis, generally recognized as a form of programmed cell death, involves the suicide and disposal of cells in response to a number of stimuli, including growth factor deprivation, antitumor drugs and ionizing radiation [13]. Apoptosis entails a broad range of morphological processes that are accompanied by membrane blebbing, nuclear fragmentation, cell shrinkage, chromosomal DNA fragmentation, and chromatin condensation [14]. Activation of apoptotic pathways plays a critical role in suppressing many types of tumoral cells, which can be served as potential anti-cancer strategies [15, 16].

Autophagy is a basic catabolic process through which damaged cytoplasmic constituents and organelles are delivered to lysosomes for proteolysis to maintain energy homeostasis [17]. Increasing evidence has been presented that autophagy plays a crucial role in tumor suppression [18]. Findings from a number of reports directed at assessing natural extracts or traditional herbs for use in cancer treatment have revealed that their cytotoxic effects involve the induction and regulation of autophagy [19, 20]. Autophagy can serve as a means to promote cell survival or, contrarily, cell death in tumor cells treated with chemotherapeutic agents [21]. The pro-survival role of autophagy contributes to cytoprotective events that help cells to maintain energy levels and survival, while its pro-death role results in the death of cancer cells [22]. These opposing functions of autophagy might be closely linked to tumor suppression, however the exact mechanisms remain unknown.

Recent findings have revealed that the c-Jun NH_2_-terminal kinase (JNK), a stress-responsive kinase, plays a vital role in the regulation of cell growth, differentiation, apoptosis, tumorogenesis, and other signaling pathways [23]. JNK is an important mediator of chemical-induced cell death and the JNK signaling pathway is required for the induction of apoptosis through a number of stress stimuli, including inflammatory cytokines, growth factors, and chemotherapeutic agents [24, 25]. In addition to these modulatory effects upon apoptosis, JNK also appears to be involved in the regulation of autophagy [26]. Of particular relevance to this report are the findings that JNK activation has been shown to be important in the regulation of autophagy induced by several antitumor agents [27, 28]. Bcl-2, the well-characterized apoptosis guards, appears to be important factors in autophagy, which represents a molecular link between autophagy and apoptosis [29]. In our study, the data indicated the underlying molecular mechanisms of vitexin's cytotoxic effects were related to apoptosis and autophagy via activation of JNK signaling pathway.

## RESULTS

### Vitexin inhibited the viability of SK-Hep1 and Hepa1-6 cells

**Figure 1:**
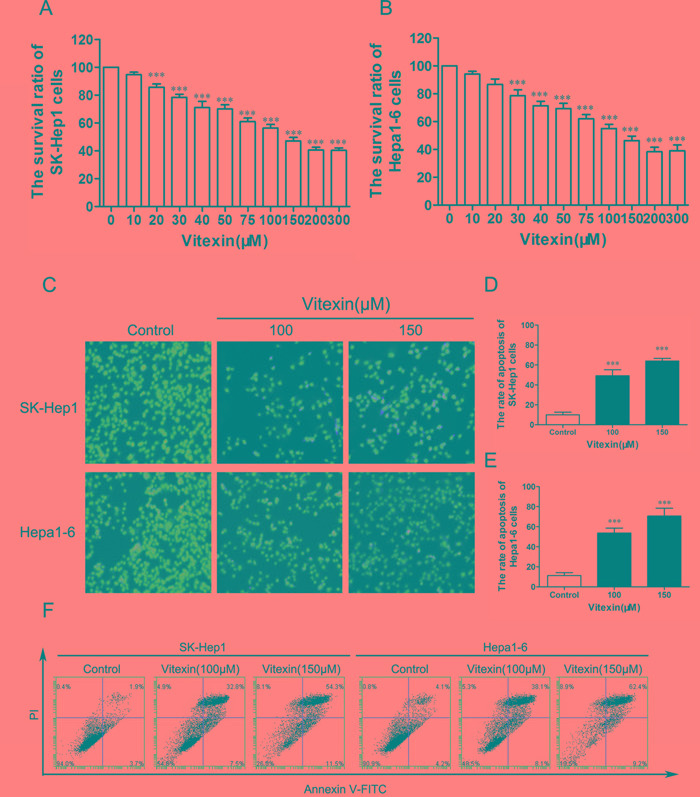
Vitexin inhibited the viability of SK-Hep1 and Hepa1-6 cells **A.** Effects of vitexin on the survival rate of SK-Hep1 cells as measured by MTT assay. **B.** Effects of vitexin on the survival rate of Hepa1-6 cells as measured by MTT assay. **C.** SK-Hep1 and Hepa1-6 cells were treated with vitexin at 100 or 150 μM for 24 h and stained with PI to reveal cell apoptosis using fluorescence microscopy (magnification ×100). **D.** The histograms indicated the rate of apoptosis of SK-Hep1 cells. **E.** The bars showed the rate of apoptosis of Hepa1-6 cells. Results shown are the mean ± standard deviation (SD) of 3 experiments. **F.** Cell apoptosis of SK-Hep1 and Hepa1-6 cells were measured with Annexin V-FITC after treated with vitexin (100 or 150 μM) for 24 h. *P<0.05, **P<0.01 and ***P<0.001 compared with control.

The effects of vitexin on cell viability in human SK-Hep1 cells and mouse Hepa1-6 cells were examined in response to increasing concentrations of vitexin for 24 h. Vitexin exerted a dose-dependent inhibitory effect on the viability of SK-Hep1 and Hepa1-6 cells (Figure 1A, 1B). Compared with the control group, the cell viability of the vitexin group at a concentration of 20 μM was significantly decreased to 85.7% in SK-Hep1 cells (*P*<0.001, Figure 1A) and to 78.7% in Hepa1-6 cells in response to 30 μM of vitexin (*P*<0.001, Figure 1B). SK-Hep1 and Hepa1-6 cells treated with 100 μM vitexin for 24 h inhibited cell viability by 56.3% and 55%, respectively, while 150 μM vitexin resulted in 47.0% and 46.3%, respectively, with both of these effects being significantly decreased as compared with the control group (100%, *P*<0.001, Figure 1A, 1B). In addition, IC_50_ values in SK-Hep1 and Hepa1-6 cells were approximately 100 μM, indicating that cell viability was significantly decreased at 100 μM concentration of vitexin treatment.

### Effects of vitexin on the induction of apoptosis in SK-Hep1 and Hepa1-6 cells

To detect whether vitexin inhibited the viability by inducing apoptosis in liver cancer cells, apoptosis was determined by flow cytometry with Annexin V-FITC/PI Staining. Both SK-Hep1 and Hepa1-6 cells were treated with 100 or 150 μM vitexin for 24 h. As shown in Figure 1C, dose-dependent increase of apoptosis cells was observed in SK-Hep1 and Hepa1-6 cells following vitexin treatment. The proportions of PI stained cells in the control, low (100μM) and high (150μM) concentration of vitexin groups were 11.3%, 49.3% and 64.0% in SK-Hep1 cells (*P*<0.001, Figure 1D) and 10.0, %, 53.7% and 70.7% in Hepa1-6 cells (*P*<0.001, Figure 1E). Furthermore, when the dose of vitexin increased from 100μM to 150 μM in both cell lines, the percentage of Annexin V-PI double stained cells increased, which also indicated an increase of apoptosis (Figure 1F).

**Figure 2:**
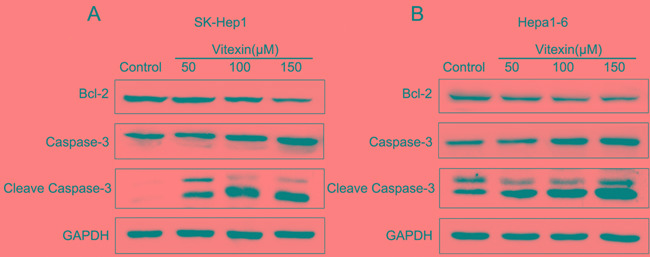
Effects of vitexin on the induction of apoptosis in SK-Hep1 and Hepa1-6 cells **A.** and **B.** Expression of apoptosis-associated proteins Bcl-2, Caspase-3 and Cleave Caspase-3 were determined by western blot after treatment with different concentrations of vitexin for 24 h in both cell lines. SK-Hep1 and Hepa1-6 cells were cultured in DMEM medium containing 50, 100, or 150 μM vitexin for 24 h. Serum-free was used as the control. The experiments were repeated 3 times.

To further evaluate the molecular mechanisms of apoptosis induced by vitexin, we explored the expression of apoptosis-related proteins in response to different concentrations of vitexin for 24 h. As shown in Figure 2A, 2B, when compared with the control group, the activity of Caspase-3 in SK-Hep1 and Hepa1-6 cells gradually increased by treatment with vitexin, while the anti-apoptosis protein Bcl-2 was down-regulated in a dose-dependent manner. The expression of Cleave Caspase-3 was also dose-dependently activated with vitexin treatment in both cell lines, which is consistent with the Caspase-3 activation. Together, the results suggested that vitexin induced apoptosis in liver cancer cells.

### Effects of vitexin on the inhibition of autophagy in SK-Hep1 and Hepa1-6 cells

**Figure 3:**
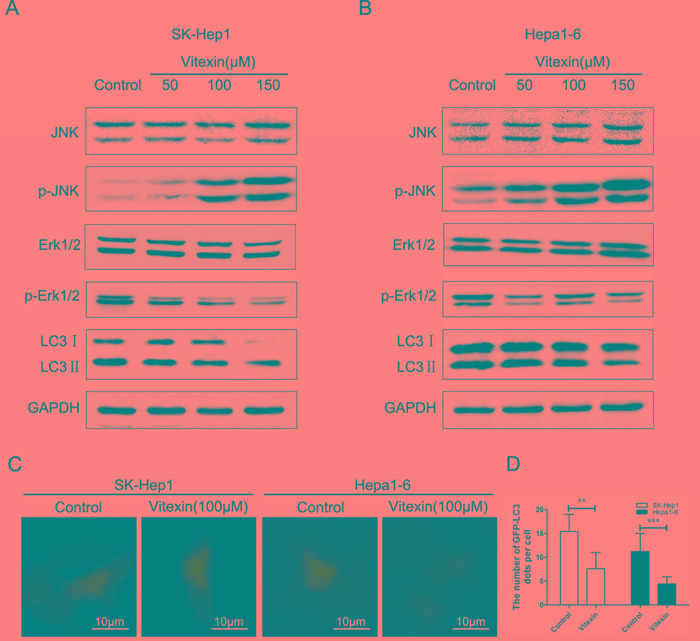
Effects of vitexin on the inhibition of autophagy in SK-Hep1 and Hepa1-6 cells **A.** and **B.** Effects of vitexin on autophagy-related protein LC3II expression levels as well as MAPK signaling pathways as detected by western blot in SK-Hep1 and Hepa1-6 cells. Cells were treated with 50, 100 or 150 μM vitexin for 24 h. Serum-free was used as the negative control. **C.** Immunofluorescence of SK-Hep1 and Hepa1-6 cells as detected by the accumulation of autophagic vacuoles and quantified by the number of GFP-LC3 dots (magnification ×400, scale bar=10 μm). Green dots represent autophagosomes. **D.** The bars showed that the number of GFP-LC3 puncta per cell in SK-Hep1 and Hepa1-6 cells. Results shown are the mean ± standard deviation (SD). *P<0.05, **P<0.01 and ***P<0.001 compared with control.

To determine whether the inhibition of autophagy was regulated by vitexin in SK-Hep1 and Hepa1-6 cells, levels of the proteins in autophagy pathway were measured by western blot. As shown in Figure 3A, 3B, the protein levels of LC3II decreased markedly in a dose-dependent manner following vitexin treatment for 24 h in both SK-Hep1 and Hepa1-6 cells. To further explore autophagy suppressed by vitexin, we examined the localization of LC3 to autophagosome formation as assessed through GFP-LC3 expression. SK-Hep1 and Hepa1-6 cells were transfected with an LC3-GFP plasmid and treated with 100 μM vitexin for 24 h. Fluorescent microscopy was then used to investigate intracellular GFP-LC3 puncta number. As shown in Figure 3C, GFP-LC3 puncta formation observed as punctate dots of green fluorescence after 24 h of vitexin treatment were significantly decreased in SK-Hep1 and Hepa1-6 cells when compared to that of controls. After treatment with vitexin, the number of GFP-LC3 dots in SK-Hep1 cells was decreased to 7.6% versus 15.4% in their control (*P*<0.01, Figure 3D), while Hepa1-6 cells was decreased to 4.4% versus 11.2% in their control (*P*<0.001, Figure 3D). These results demonstrated that LC3-associated autophagosome formation was inhibited by vitexin treatment.

### Activation of autophagy inhibited vitexin-induced apoptosis in SK-Hep1 and Hepa1-6 cells

**Figure 4:**
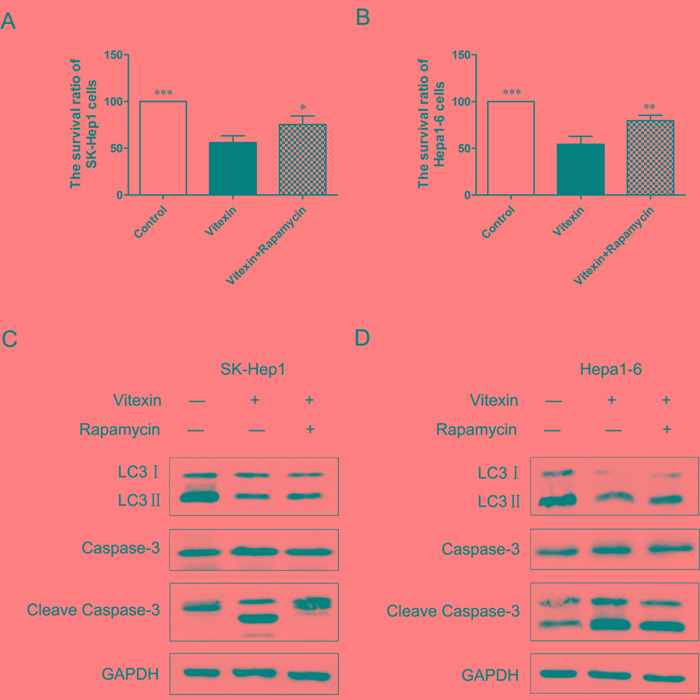
Activation of autophagy inhibited vitexin-induced apoptosis in SK-Hep1 and Hepa1-6 cells **A.** and **B.** SK-Hep1 and Hepa1-6 cells were treated for 24 h with 100 μM vitexin, in the presence or absence of 40 nM rapamycin. Cell survival rates were measured by MTT assay. Results shown are the mean ± standard deviation (SD). *P<0.05, **P<0.01 and ***P<0.001 compared with control. **C.** and **D.** SK-Hep1 and Hepa1-6 cells were treated with 100 μM vitexin for 24 h, in the presence or absence of rapamycin. LC3II, Caspase-3 and Cleave Caspase-3 densitometric analysis were determined using western blot.

In order to confirm the protective role of autophagy against vitexin-induced apoptosis, we investigated the effects of activating autophagy with use of rapamycin, a specific autophagy activator. The combination of rapamycin and vitexin significantly increased the survival rate of SK-Hep1 cells to 75.3% versus 56% with vitexin alone (*P*<0.05, Figure 4A) and Hepa1-6 cells to 79.7% versus 54.3% with vitexin alone (*P*<0.01, Figure 4B). Compared with groups treated with vitexin alone, groups of vitexin plus rapamycin showed an increase in LC3II levels which was inhibited by vitexin (Figure 4C, 4D). In contrast to that, the levels of apoptosis-related proteins Caspase-3 and Cleave Caspase-3 were dramatically decreased in the presence of vitexin and rapamycin, compared with vitexin alone (Figure 4C, 4D). These results substantiated that autophagy activated by rapamycin can suppress vitexin-induced apoptosis in SK-Hep1 and Hepa1-6 cells.

### Vitexin induced apoptosis and suppressed autophagy through MAPK signaling pathway in SK-Hep1 and Hepa1-6 cells

Findings from a number of reports have indicated that vitexin induces activation of MAPK pathways, which is the central regulator of cell growth and regulates apoptosis and autophagy in response to stress conditions [30]. Accordingly, JNK activation induced by vitexin was examined to assess the role of this signal-transduction by western blot. Figure 3A, 3B showed that the levels of phosphorylated JNK (p-JNK) were remarkably increased in a dose-dependent manner in response to vitexin treatment. In contrast to that of p-JNK, the levels of phosphorylated forms of Erk1/2 (p-Erk1/2) were significantly decreased in SK-Hep1 and Hepa1-6 cells following vitexin treatment (Figure 3A, 3B). No significant changes in total JNK and Erk1/2 expression were found for either cell line in response to vitexin treatment in different concentrations.

**Figure 5:**
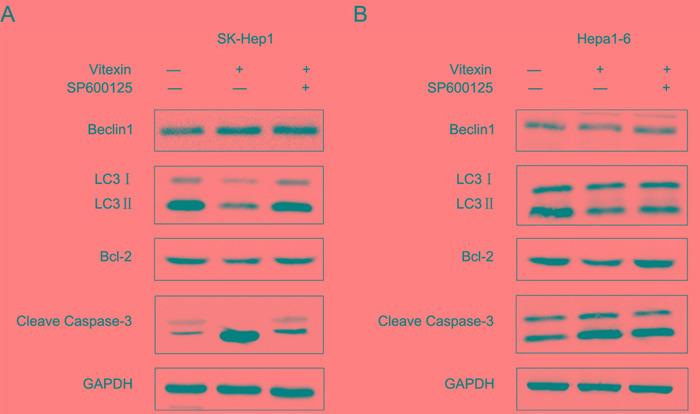
Vitexin induced apoptosis and suppressed autophagy through MAPK signaling pathway in SK-Hep1 and Hepa1-6 cells **A.** and **B.** After 24 h treatment with 100 μM vitexin with or without 10 μM SP600125, the autophagy and apoptosis-associated proteins expression of Beclin1, LC3, Bcl-2 and Cleave Caspase-3 were detected using western blot in SK-Hep1 and Hepa1-6 cells. GAPDH was used as an internal control to normalize the amount of proteins applied in each lane.

To identify whether the activation of JNK signaling pathway was related to apoptosis and autophagy mediated by vitexin in HCC cell lines, the specific JNK inhibitor (SP600125) was used to block JNK activity. We found that cotreatment with SP600125 (10 μM) reversed LC3II reduction caused by vitexin (100 μM) in both cell lines (Figure 5A, 5B). In SK-Hep1 cells, protein levels of the cleaved form of Caspase-3 were increased with vitexin treatment, but attenuated in the combined vitexin (100 μM) and SP600125 (10 μM) condition (Figure 5A), as well as in Hepa1-6 cells (Figure 5B). The results also showed that pretreatment with the JNK inhibitor reversed the down regulation of Bcl-2 by vitexin in SK-Hep1 and Hepa1-6 cells (Figure 5A, 5B). These results, which demonstrated that SP600125 enhanced vitexin-inhibited autophagy and reduced vitexin-induced apoptosis in SK-Hep1 and Hepa1-6 cells, indicated that JNK signaling pathway activated by vitexin contributed to the inhibition of autophagy and induction of apoptosis.

### Effects of vitexin on tumor growth suppression *in vitro* and *in vivo*

**Figure 6:**
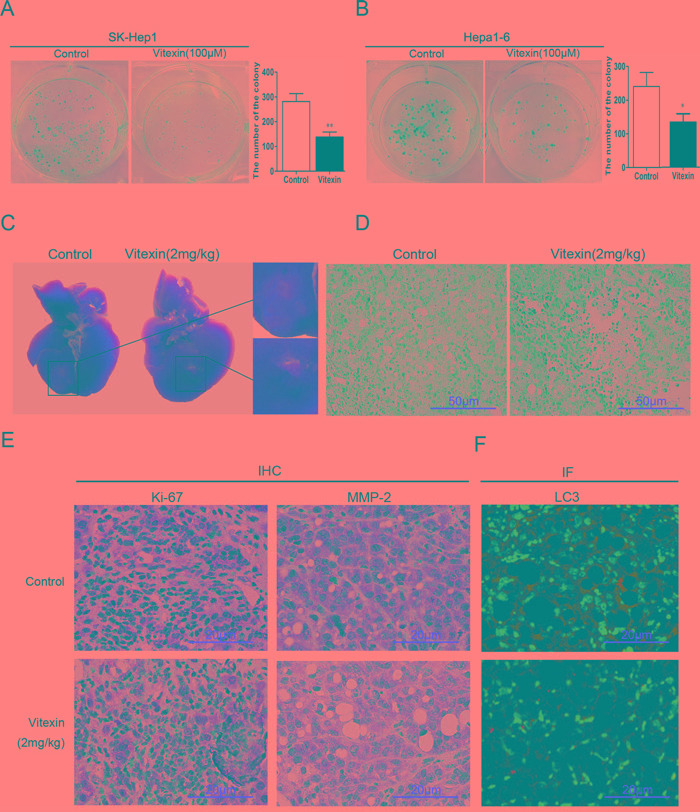
Effects of vitexin on tumor growth suppression *in vitro* and *in vivo* **A.** and **B.** Colony formation assay was developed to measure the tumor inhibitory effects of vitexin in HCC cell lines. The bars showed the number of the colony in both cell lines. **C.** Tumor volumes were measured and examined histologically after subcutaneous injection of Hepa1-6 cells into the left liver lobes of mice following 4 weeks treatment of vitexin (2mg/kg) or 0.1% DMSO. **D.** Hematoxylin and eosin (H&E) staining was used to observe histopathologic changes in mouse liver (magnification ×100, scale bar=50 μm). **E.** The expression of Ki-67 and MMP-2 were examined by immunohistochemistry from tumor samples with or without vitexin treatment (magnification ×200, scale bar=20 μm). **F.** Immunofluorescence was used to determine the expression of LC3 in the tumor samples with or without vitexin treatment (magnification ×200, scale bar=20 μm).

Colony formation assay was developed to further examine the capacity of vitexin to inhibit growth *in vitro* within liver cancer cells. In Figure 6A and 6B, we showed that the colony forming abilities of SK-Hep1 and Hepa1-6 cells were largely inhibited by vitexin. We also observed antitumor effects of vitexin as demonstrated *in vivo* within the established liver cancer C57BL/6 mouse model. As compared with tumor formation obtained in the non-vitexin treated group, vitexin pretreatment resulted in a significant suppression in tumor size (Figure 6C). Results of hematoxylin and eosin (H&E) staining demonstrated that tumor tissue in vitexin treated group showed more pathological changes of apoptosis and necrosis compared to that in non-vitexin treated group (Figure 6D). Immunohistological staining showed that expression of Ki-67 and MMP-2, which are regarded as indices evaluating proliferation and invasion of tumor, were decreased in the vitexin treatment group (Figure 6E). Immunofluorescence analysis, which was then used to determine the expression of LC3 in the tumor samples, revealed that a low-expression of LC3 was presented in the vitexin pretreatment group versus non-vitexin group (Figure 6F). In this way, results obtained from *in vitro* and *in vivo* experiments indicated that vitexin exerted an inhibitory effect on HCC tumor growth.

## DISCUSSION

Natural products can provide a favorable resource for cancer prevention and treatment [31]. Vitexin has been widely used for treatment of asthma and coughs and has also been shown to exert anti-oxidative, anti-inflammatory and analgesic effects as well as being an anti-cancer agent [32, 33]. With regard to this latter function, vitexin has been shown to possess antitumor effects within various cancer cell lines [30, 34]. The tumor inhibitory effects of vitexin have been shown to be associated with cell proliferation, cell growth, apoptosis and dysregulation [35, 36]. Here we showed that vitexin inhibited the viability of hepatocellular carcinoma in SK-Hep1 and Hepa1-6 cells as well demonstrating antitumor activity *in vivo* with an established liver cancer mouse model. Moreover, we reported some of the potential mechanisms of vitexin's antitumor effects involving apoptosis, autophagy and JNK MAPK signaling pathways.

Apoptosis is a self-suicide process by which damaged, mutant and aged cells are eliminated through internal molecular mechanism. Disruption of this programmed cell death process can result in a variety diseases including cancer [37, 38]. Findings from a number of studies have indicated that apoptosis is vital to tumor growth and may result in the induction of death in cancer cells after chemotherapeutic agents [39, 40]. Bcl-2 is considered as an anti-apoptotic protein which binds to the mitochonrial membrane and protects against cellular death by preventing the release of cytochrome c [41]. Bcl-2 is crucial to the downstream effectors of caspase-3 activation and determination of whether cells will proceed or not with apoptosis [42]. Caspase-3 represents a critical factor that best correlates with apoptosis and is responsible for the proteolytic cleavage of many vital proteins. In this study, we observed that the vitexin suppressed liver cancer cell viability and induced apoptosis by decreasing Bcl-2 protein expression while activating Caspase-3 and its cleavage form. These findings suggest that vitexin-induced apoptosis contributes to the suppression of liver cancer cell viability, which may thus be a promising chemopreventive agent for cancer treatment. It has been reported that Vitexin 6 (VB6) induced apoptosis and autophagy in human breast cancer cell line T-47D. The levels of Beclin-1 and LC3-II gradually increased after VB6 treatment [30]. The regulation of autophagy may vary according to the types of cancer and the crosstalk between autophagy and apoptosis is also needed to be clear.

Autophagy also plays an essential role in tumor development and is implicated in the treatment of a variety of diseases, due to its involvement with metabolic stress, damnification and tumorigenesis [43, 44]. A number of natural products, such as Oroxylin A, have proved to be effective chemotherapeutic agents through their capacity to inhibit autophagy [45]. The conversion of LC3I to LC3II contributes to the formation of autophagosome and thus LC3II is usually considered as a marker of autophagy [46, 47]. In the present study, vitexin treatment markedly decreased LC3II protein levels and the number of GFP-LC3 dots compared to the control group, suggesting that vitexin inhibited autophagy in HCC cell lines. Beclin1 is also necessary for the induction of autophagy and regulates the initiation and formation of autophagosome by activation of proteins of the autophagic complex, including Beclin1 [48], Vps34 [49], and Bcl-2. To further assess the role of autophagy on HCC, rapamycin (an inhibitor of mTOR) was administrated to the HCC cell lines. Cotreatment with rapamycin increased the survival ratio of SK-Hep1 and Hepa1-6 cells which suggested a protective role of autophagy in vitexin treated HCC cells. The down-regulation of LC3II expression by vitexin was reversed by rapamycin, as well as the up-regulation of Caspase-3 and Cleave Caspase-3. These results indicated that autophagy exerted a pro-survival effect in HCC cell lines and vitexin suppressed tumor growth through inhibiting autophagy and increasing apoptosis.

It has been reported that MAPKs are involved in a variety of cellular signal transduction pathways, including apoptotic cell death, inflammation and oncogenesis [50]. JNK MAPK is mainly considered as a stress-activated protein related to apoptotic cell death, whereas Erk1/2 behaves mainly as a mitogen-activated proliferation factor. Activation of JNK and inhibition of Erk1/2 MAPK play critical roles in the antitumor activity of natural compounds [51]. The natural product, evodiamine, has been demonstrated to induce apoptosis through activation of JNK pathways [52]. Puissant et al [53] reported that resveratrol induced autophagy in chronic myeloid leukaemia via JNK-dependent p62 expression. JNK has been reported to be involved in various stimulation-induced autophagic events, including endoplasmic reticulum stress [54], insulin-like growth factor-1 treatment and exposure to tumor necrosis factor α [55]. In the present study, we found that JNK MAPK signaling pathway was activated, but the levels of Erk1/2 MAPK were decreased by vitexin treatment. Such changes suggest that MAPK contributes to the regulation of autophagy and apoptosis in liver cancer cells. With the use of SP600125, a specific JNK inhibitor used to block JNK activity, a reduction in lipopolysaccharide-induced apoptosis in MC3T3-E1 cells was observed [56]. In our study, we observed that SP600125 pretreatment enhanced vitexin-reduced autophagy and inhibited vitexin-induced apoptosis in SK-Hep1 and Hepa1-6 cells, suggesting that the activation of JNK signaling plays a crucial role in the induction of apoptosis and inhibition of autophagy in liver cancer cells by vitexin treatment.

In the present study, results of colony formation assay showed that vitexin inhibited the growth of HCC cells, we further explored the antitumor effects of vitexin on HCC xenograft models *in vivo*. Notably, we observed that vitexin inhibited tumor growth *in vivo* and induced apoptosis and necrosis, which is similar to that of previous study [11]. Meanwhile, vitexin decreased the expression of Ki67 and MMP-2, the most commonly used markers for tumor proliferation and invasion. In summary, our results suggest that vitexin possesses potent antitumor efficacy through suppressing autophagy to induce apoptosis, which is mediated by activation of JNK signaling pathway.

## MATERIALS AND METHODS

### Reagents and antibodies

DMEM medium and FBS were purchased from Gibco (Grand Island, NY). Vitexin, obtained from SelleckInc (USA), was dissolved in dimethylsulfoxide (DMSO; Sigma-Aldrich) at a concentration of 300 mmol/L stock solution and stored at -20°C. MTT (3-[4, 5-Dimethylthiazol-2-yl]-2, 5-diphenyl tetrazolium bromide), DAPI (6-diamidino-2-phenylindole dihydrochloride), and the Annexin V-FITC/PI Staining Apoptosis Detection Kit were purchased from Sigma-Aldrich. Rapamycin and SP600125 (JNK inhibitor) were purchased from Selleck Inc (USA). Antibodies for Beclin1, LC3, JNK, p-JNK, Erk1/2, p-Erk1/2, Bcl-2, Caspase-3, Cleave Caspase-3, Ki-67, MMP-2, GAPDH and HRP-conjugated secondary antibodies were purchased from Cell Signaling Technology Inc (USA). All other reagents were purchased from Sigma-Aldrich or as indicated.

### Cell culture and treatment

SK-Hep1 cells (a human liver cancer cell line) and Hepa1-6 cells (a mouse liver cancer cell line) were purchased from the Institute of Biochemistry and Cell Biology of the Chinese Academy of Sciences (Shanghai, China). Cells were incubated in Dulbecco's modified Eagle's medium (DMEM) with 10% fetal bovine serum (FBS) and 1% penicillin streptomycin (100 U/mL) at 37°C under 5% CO_2_.

SK-Hep1 and Hepa1-6 cells cultured in DMEM were plated at a density of 2 × 10^5^ cells/mL in 6-well plates and divided into four groups: (1) Control group: Cells without treatment were served as the control condition. (2) Vitexin group: Cells were treated with different concentrations of vitexin for 24 h. (3) Rapamycin group: Cells were treated with 100 μM vitexin and 40 nM rapamycin for 24 h. (4) SP600125 group: Cells were treated with vitexin (100 μM) combined with SP600125 (10 μM) for 24 h.

### Cellular viability analysis

The effects of vitexin on SK-Hep1 and Hepa1-6 cells viability were assessed with use of a MTT kit (Sigma-Aldrich). Cells were plated into 96-well plates (5 × 10^4^ cells/well) and after 24 h of incubation at 37°C, then treated with 0, 10, 20, 30, 40, 50, 75, 100, 150, 200, or 300 μM vitexin for 24 h. Fresh medium and 20 μL MTT solution (5 mg/mL) was added to each well and cultured for 4 h at 37°C and 5% CO_2_. After incubation, the medium was removed and 200 μL DMSO was added to each well to detect the absorbance. The OD values were determined at a wavelength of 570 nm. IC_50_ value represented the concentration of vitexin required to inhibit cell viability by 50% relative to untreated cells and were calculated by the logit method.

### Cell apoptosis using annexin V-FITC/PI staining

The cells were placed in a 6-well plate and incubated with vitexin (100 or 150 μM) for 24 h. Cells without any deal served as control. Cells were harvested, washed in PBS and resuspended in annexin-binding buffer. Then 5 μl Annexin V-FITC and 10 μl PI Staining Solution were added and cells were cultured at room temperature in the dark for 20 min. Apoptotic cell death was analyzed after DAPI staining by flow cytometry. For quantification and distribution, the rate of cell apoptosis was determined under fluorescent microscope using 100× magnification with an average from five different fields used to calculate this quantified value.

### Western blot analysis

Protein samples extracted from HCC cell lines were lysed with SDS-PAGE sample buffer. Protein samples were separated and transferred onto nitrocellulose membranes by 10% or 12% SDS-PAGE. The membranes were blocked with TBS containing 5% nonfat milk and then incubated with antibodies (Beclin1, LC3, JNK, p-JNK, Erk1/2, p-Erk1/2, Bcl-2, Caspase-3, Cleave Caspase-3 and GAPDH) at 4°C overnight. After washing, the membranes were incubated with HRP-conjugated antiIgG at room temperature for 1 h. Signal detection was performed with an ECL system (Millipore, Billerica MA, USA).

### Cell transfection and fluorescence microscopy

SK-Hep1 and Hepa1-6 cells were transfected with the GFP-LC3 plasmid using Lipofectamine 3000™ (Invitrogen) according to the manufacturer's guidelines. After cells had been treated with or without 100 μM vitexin, the puncta formation of GFP-LC3 was determined under fluorescent microscopy (Olympus BX5). Cells were considered to have accumulated autophagosomes when five or more puncta were counted. This criteria was established as the small number of mock-treated cells rarely displayed more than one to four puncta.

### Colony formation assay

The single-cell suspensions of SK-Hep1 and Hepa1-6 cells were placed in a fresh 6-well plate in triplicate for each group in medium containing 10% FBS at 37°C. After 24 h, the cell lines were replaced with new medium together with 100 μM vitexin. Drugs were washed out at 48 hours after treatment, and fresh 10% FBS medium was added. After 14 days, cells were fixed with methanol and stained with 0.1% crystal violet. Dishes were washed with distilled water, and visible colonies were manually counted.

### Animal experiments *in vivo*

Male C57BL/6 mice were purchased at 6-7 weeks of age from the experimental animal center of the PLA Military Medical Science Academy. Animal experiments were performed according to the guidelines of the National Institute of Health (NIH). A mouse xenograft model of HCC was established as described previously [57]. Hepa1-6 cells (2 × 10^7^) were re-suspended in 200 μl serum-free culture medium and were subcutaneously injected into the left liver lobes of C57BL/6 mice [58]. The mice were divided into groups of 3 mice. Tumor growth was examined weekly for 5 weeks. When the tumors became palpable, animals were treated with vitexin (2 mg/kg) or 0.1% DMSO (control) by intraperitoneal injection daily for 4 weeks. Then the mice were sacrificed, necropsies were performed and the tumor tissues were harvested. Tumor volumes of the control and vitexin group were measured and examined for histological analysis.

### Hematoxylin and eosin

Samples from the liver tumor tissues were fixed in 4% paraformaldehyde for 24 h, dehydrated and embedded in paraffin. Five micrometer-thick sections were cut from each paraffin embedded tissue and stained with hematoxylin and eosin (H&E) for histopathologic examination under a light microscope.

### Immunohistochemical staining

Tumor tissues samples were fixed in 4% paraformaldehyde for 24 h, dehydrated, embedded in paraffin, and sectioned at a 5-μm thickness. Tissue sections were deparaffinized and rehydrated with xylene and alcohol of gradient concentrations, then cultured in 5 mmol/L citrate buffer (pH 6.0) for 15 minutes to retrieve internal antigens by microwave. After blocking endogenous peroxidase activity by incubating in 3% H_2_O_2_ for 15 min, sections were blocked with 5% goat serum, and incubated with antibodies (Ki-67 and MMP-2) at 4°C overnight. Sections were incubated at room temperature for 60 min with the HRP-conjugated secondary antibody, then DAB solution was used. Counterstaining was performed with hematoxylin.

### Immunofluorescence staining

After retrieving internal antigens and blocking endogenous peroxidase activity as described above, tumor sections were treated with 5% goat serum and incubated with LC3 antibody at 4°C overnight. Sections were incubated with 20μl fluorophore-conjugated secondary antibody for 1 h at room temperature before imaging. After washing with PBS, sections were counterstained with DAPI (10 ng/mL) for 10 min. A fluorescence microscope was used for LC3 co-localization analysis.

### Data analysis and statistical procedures

The results were expressed as the mean ± standard deviation (SD) of independent experiments. The data were analyzed using one-way analysis of variance (ANOVA) to determine statistically significant differences among groups. Differences were considered statistically significant for values of *P*<0.05. All statistical analysis were performed with SPSS 19.0 and GraphPad Prism Version 5.0 softwares.
